# Notes and Comments

**Published:** 1922-12

**Authors:** 


### TUBERCULOSIS IN DOMESTIC ANIMALS.

THE part played by domestic animals, such as cats and dogs, in the dissemination of tuberculosis in man has received little attention. It certainly does not deserve great attention, for infected human beings and cattle are much more important sources. But now and then tuberculosis in domestic animals has been associated with tuberculosis in man, and it is well not to ignore this source of infection altogether. Professor Lydia Rabinowitsch-Kempner has recently reported on investigations carried out by herself and Jost on cats and dogs. Among 31 dogs suspected of suffering from tuberculosis, 19 were found to be definitely tuberculous, and in as many as 16 the tubercle bacilli belonged to the human type. Among six cats, three were found to harbour the human type and two the bovine type of tubercle bacillus. Most of these animals were probably infected by man, for they had not been fed on milk, nor been in contact with tuberculous cattle. The fact that the tuberculous dogs belonged to breeds which are most petted also pointed to infection by consumptives. The disease having passed from man to domestic animals, the chances of the reverse process cannot be lightly dismissed. Incidentally this publication throws light on conditions during and after the war. Almost all the dogs examined in Berlin during 1920 and1921 were young; most of the older ones had been eaten.

### THE MEETING OF THE WOMEN'S INTERNATIONAL ASSOCIATION.

THE first congress of the Medical Women's International Association was held in Geneva on Sept. 4-6 of this year, and was the occasion for taking stock of the numbers and status of women doctors throughout the world. The task of analysing their status is naturally somewhat invidious; the echoes of the outcries against them are still too loud to permit a dispassionate judgment of the reasons and justifications for the opposition which they encountered a generation ago. We are on safer grounds in a discussion of their numbers, and it is interesting to note that of the sum total of about 10,000 women doctors, approximately 6,000 are settled in the U.S.A. and 2,000 in England. This leaves only about 2,000 distributed over 71 other countries, and it is therefore evident that, apart from the two great Anglo-Saxon countries, the dilution of women in the medical profession is almost homeopathic. Women doctors appear to concentrate in the towns, and of 140 settled in Austria, as many as 120 are to be found in Vienna. With regard to their activities, it is generally assumed that many marry and cease to practise, but hitherto little accurate information has been available on this point. Some light is thrown on it by a recent analysis of the careers of women doctors in Denmark, where, since 1885, as many as 116 women have qualified. Of this total, eight have died, seven have married and given up practice, 33 have not yet started practice, nine have settled in practices abroad, and 59 have settled in practice in Denmark. Of these 59, 24 have married, as many as 14 of these marriages being with men doctors. It will thus be seen that of the total of 116, only 35 are practising independently in Denmark. These figures suggest that, though many women may pass all the necessary examinations, they seldom become independent medical practitioners, and the fears that they would compete with and supplant men doctors, dragging down the standard of remuneration, if not that of efficiency, have evidently been much exaggerated. At Geneva papers were read on the campaign against venereal disease, on sex education and infant welfare. There could not have been a better choice of subjects. The part played, for example, by women in the recent campaign against venereal disease in England is far greater than is generally known or acknowledged. It is largely due to women, many of them doctors, that we have come to regard as vile and intolerable the unwritten law that a man's moral and sexual code should be more lax than a woman's; of the many predisposing causes of venereal disease, this has been one of the most potent. The Medical Women's International Congress concluded, we are glad to note, with a banquet. Congresses at all times are rather dull and serious concerns, and in giving the lighter touch to their meeting, the organisers of this Congress showed an appreciation of the wisdom of combining social with sociological and scientific interests. The Association meets again in five years.

---

### OUTBREAK OF SMALLPOX IN LONDON.

A SERIOUS outbreak of smallpox has occurred in London. It began with the discovery on October 27 of a case at St. Andrew's Hospital, Bromley-by-Bow, when the rash appeared on the 24th on an inmate admitted from the Poplar Institution (formerly called the Poplar Workhouse). On the same evening vaccination of the immediate "contacts" was begun and subsequently the staff and nearly all of the inmates were revaccinated. On the 28th five cases were discovered in the Poplar Institution. The Institution was at once isolated, the cases of smallpox were removed to hospital and disinfection carried out. The dates of onset showed that these five patients had been infected from one source from about October 13 to 15. Inquiry was made for a person who had fallen ill about that date. One was found and proved to have the rash of smallpox. Whence he contracted the disease was not known. Other cases followed.

Altogether 38 cases occurred, with 11 deaths, including four outside the Institution in persons connected with it. The outbreak now appears to be over. Thanks to the alertness of the medical staff, a much more extensive outbreak has been averted. All over London vaccinations are being performed. In view of the neglect of vaccination, they are very necessary. Curiously, persons who at ordinary times regard vaccination as useless abandon this view when confronted with the danger of smallpox.

The Ministry of Health has issued memoranda enjoining vaccination. Only 40 per cent. of the babies are now being vaccinated, which greatly increases the risk of an epidemic. Medical officers of health are authorised to vaccinate "contacts." But the primary responsibility for vaccination at the public expense remains with Boards of Guardians. District medical officers attending on a case of smallpox are entitled to payment from the Guardians for vaccinating any person resident in the house. The prompt vaccination of children between the ages of six months and 14 years is recommended, and in infected localities also of children below six months. In ordinary circumstances no infant should be vaccinated under the age of two months, but younger infants should be vaccinated if exposed to infection. On the receipt of a notification of smallpox the public vaccinator should offer to vaccinate all the inmates of the house. Medical officers of health are asked to examine the weekly statistical returns, so that the liability of any district to introduction of the disease shall be recognised early. Compulsory notification of chicken-pox is advised if an extensive outbreak of smallpox appears probable. Every person suffering from smallpox should be removed to the smallpox hospital. Such removal may, if required, be ordered by a justice.

---

### MUSIC AND HEALING.

UNDER this title, Mr. H. E. Wortham, the musical critic, lately drew attention in the "Morning Post" to some therapeutic experiments with music that were being reported from America. His concern chiefly was, not the details of the American treatment, but the tradition whereby, from the time of Saul, music has been held of value in certain diseases. With wit and learning, the writer resurrected some of these, which are by no means necessarily mental. Gout, for example, was deemed to yield to music in antiquity, if the proper mode were chosen. But, curiously enough, Mr. Wortham did not mention two traditions still current among musicians upon the point. It is believed by many members of orchestras that violin playing encourages abundance of hair, and also that bassoonists run to baldness. Statistics, carefully controlled, in the manner of the biometrists, should be able to decide the point; and if statistics justify the tradition, we shall be encouraged to examine the causes of an influence that remains at present obscure, despite the experiments now being made in America.

---

### CENSUS FIGURES.

THE first volume giving the results of the census taken last year has been published. It deals with London, and, for the convenience of the public is divided into three parts, the first two mainly statistical, the third containing comment. The Registrar General is making special arrangements to meet the demands of public bodies and of private individuals for information that is not contained in the published volumes. He stated to a representative of this Review that any individual needing special statistics should write to him at Somerset House, when an estimate of the cost of collating the required data would be provided. This is an arrangement, the convenience of which will be appreciated by students of public health, and is an improvement on the old days when too often official documents collected dust in a pigeon hole. In this case Mr. S. P. Vivian, the Registrar General, believes that it is of no use to spend money on taking a census if the results are not to be useful to the public.

